# Impact of milk consumption on cardiometabolic risk in postmenopausal women with abdominal obesity

**DOI:** 10.1186/1475-2891-14-12

**Published:** 2015-01-21

**Authors:** Jean-Philippe Drouin-Chartier, Josée Gagnon, Marie-Ève Labonté, Sophie Desroches, Amélie Charest, Geneviève Grenier, Sylvie Dodin, Simone Lemieux, Patrick Couture, Benoît Lamarche

**Affiliations:** Institute of Nutrition and Functional Foods (INAF), Laval University, Quebec, QC G1V 0A6 Canada

**Keywords:** Milk, Dairy, Metabolic syndrome, Blood pressure, Cardiovascular disease, Randomized controlled study, Postmenopausal women

## Abstract

**Background:**

The impact of dairy intake on cardiometabolic risk factors associated with metabolic syndrome (MetS) needs further research.

**Objective:**

To investigate the impact of milk consumption on a wide array of cardiometabolic risk factors associated with MetS (blood lipids, cholesterol homeostasis, glucose homeostasis, systemic inflammation, blood pressure, endothelial function) in postmenopausal women with abdominal obesity.

**Methods:**

In this randomized, crossover study, 27 women with abdominal obesity consumed two 6-week diets based on the National Cholesterol Education Program (NCEP), one with 3.2 servings/d of 2% fat milk per 2000 kcal (MILK) and one without milk or other dairy (NCEP). The macronutrient composition of both diets was comparable (55% carbohydrates, 15% proteins, 30% fat and 10% saturated fat).

**Results:**

The MILK diet had no significant effect on LDL-C, triglycerides, LDL size, CRP and cell adhesion molecule concentrations and on indicators of insulin sensitivity. The MILK diet reduced HDL-C, adiponectin, endothelin and fasting glucose levels as well blood pressure (all P ≤ 0.01), but those changes were comparable to those seen with the NCEP milk-free diet (all between-diet P ≥ 0.07). Finally, the MILK diet was associated with lower VLDL apolipoprotein B fractional catabolic rate (−13.4%; P = 0.04) and plasma sterol concentrations (−12.0%; P = 0.04) compared with the control NCEP milk-free diet.

**Conclusions:**

These data suggest that short-term consumption of low fat milk in the context of a prudent NCEP diet has no favorable nor deleterious effect on cardiometabolic risk factors associated with MetS in postmenopausal women with abdominal obesity.

**Electronic supplementary material:**

The online version of this article (doi:10.1186/1475-2891-14-12) contains supplementary material, which is available to authorized users.

## Background

A significant proportion of CVD events in industrialized countries is attributable to the presence of a cluster of cardiometabolic perturbations associated with metabolic syndrome (MetS) [1]. This syndrome encompasses abdominal obesity and dysregulations of glucose and insulin metabolism along with hypertension, impaired endothelial function [2], inflammation, and a typical dyslipidemic state that includes elevated plasma triglycerides (TG) concentrations, low HDL-cholesterol (C) concentrations and small dense LDL particles [3]. The dyslipidemic state seen in MetS is caused in part by increased secretion of VLDL apolipoprotein (apo) B particles and by an enhanced HDL apoA-I clearance [4]. Recent studies have suggested that MetS may also be associated with a perturbed cholesterol homeostasis characterized by increased endogenous cholesterol synthesis and decreased cholesterol absorption [5]. MetS in various populations has been shown to be quite prevalent, with values ranging from 8% to 46% depending on country, gender [6] and age [7]. The prevalence of the MetS is particularly high in postmenopausal women [8].

Diet is considered to be a key etiological determinant of MetS [9–11]. Accordingly, data from several prospective cohort studies have shown that dairy and milk consumption was associated with a lower prevalence of MetS [12, 13]. Meta-analyses of cohort studies have also shown that milk intake was associated with a reduced risk of hypertension [14, 15], but not with type 2 diabetes risk [16–18]. On the other hand, data from randomized clinical trials having assessed the impact of milk consumption on cardiometabolic risk factors are mixed and not fully consistent with data from epidemiological studies [19–24].

In order to better understand the effect of milk consumption on cardiometabolic health, we have investigated in a randomized crossover controlled study the impact of milk consumption on a large array of cardiometabolic features associated with MetS, namely blood lipids, cholesterol and glucose homeostasis, blood pressure, endothelial function, inflammation, VLDL apoB and HDL apoA-1 kinetics in postmenopausal women with abdominal obesity. We hypothesized that milk consumption as part of a prudent diet has significant beneficial effects on blood lipids, on cholesterol homeostasis as well as on blood pressure.

## Methods

### Subjects

Twenty-nine overweight postmenopausal women in good health were recruited in the Quebec City Metropolitan area. Twenty-seven of these women completed the study. Two subjects have dropped out for personal reasons that were unrelated to the dietary intervention. All participants were Caucasian of French ancestry except two women with South Asian and Chilean ancestries respectively. Subjects were initially screened on the basis of a complete physical examination and medical history. Women eligible for the study had to be less than 70 years of age, non-smokers, with absence of menses > 12 months, follicle-stimulating hormone (FSH) > 35 IU/ml, and waist circumference ≥ 88 cm. Women with a previous history of CVD, type 2 diabetes, monogenic dyslipidemia, using lipid-lowering medications, with endocrine disorders or on hormonal replacement therapy were not eligible. Exclusion criteria also included aversion for milk, particular nutritional habits such as limited or absence of dietary animal proteins, excessive alcohol consumption (>2 drinks/day) or significant weight change in the 6 months that preceded the experiment. There was no inclusion/exclusion criteria related to dairy intake as part of participants’ usual diet. MetS was defined based on the NCEP-ATP III definition for women [25], i.e. meeting 3 or more of the 5 following criteria: waist circumference > 88 cm, plasma triglycerides > 1.7 mmol/L, plasma HDL-C <1.3 mmol/L, blood pressure ≥130/85 mm Hg and fasting glucose >6.1 mmol/L. The study protocol was explained to the participants, who gave their written informed consent. The protocol was approved by the Clinical Research Ethics Committee of Laval University. This trial was registered at clinicaltrials.gov as NCT01163773.

### Experimental design and dietary intervention

A randomized crossover design was used to investigate the impact of milk consumption on cardiometabolic features of MetS. The protocol first included a 4-week stabilization run-in period during which participants were instructed to comply to the NCEP Phase 1 prudent dietary recommendations [25]. Participants then consumed in random order a diet from which approximately 20% of calories came from partly skimmed milk (2% fat) and a control NCEP milk-free diet. Both pre-defined regimens were consumed under isocaloric conditions for periods of 6 weeks each separated by a wash-out period of 6 to 8 weeks, during which the NCEP Phase 1 dietary guidelines were reinforced. During the run-in and washout periods, women were asked to maintain their weight constant. Also, dairy products consumption was not formally excluded. In that context, women were consuming substantially more milk during the MILK phase (3.2 serv/d) and substantially less milk during the NCEP control phase (0 serv/d) than during the run-in (1.4 serv/d) and wash-out periods (1.3 serv/d).

All meals were provided to participants during the dietary controlled phases of the study. Lunch was consumed at the Clinical Investigation Unit every weekday under the monitoring of registered dieticians while breakfast and dinner on weekdays and all meals on weekends were packed and consumed at home. Subjects were asked to eat all the food and only the food that was provided to them. They were provided with a daily reminder sheet on which they were required to check all food items consumed and to report any deviation from the diet, as well as any illness or use of medication. Analysis of these checklists indicated that compliance to the experimental diets was excellent (>98%).

Usual energy and dietary intakes were assessed at screening using a validated self-administered food frequency questionnaire (FFQ) [26]. Participants began the study at a pre-defined energy level that was closest to the mean between their energy intake estimated by the FFQ and by the Harris-Benedict formula. Weight was monitored every weekday before lunch, and the energy level was adjusted if body weight had fluctuated from baseline by more than 1.0 kg. Consumption of alcohol as well as of vitamin supplements and natural health products during each experimental period was forbidden. Consumption of caffeinated beverages such as diet soft drinks (12-oz or 355 ml), coffee and tea (8-oz or 237 ml) was limited to a maximum of 2 drinks/d.

Participants and staff could not be blinded to the experimental procedures due to the inclusion of milk in the MILK phase. However, all laboratory analyses were undertaken in a blinded fashion. Participants were asked to maintain their usual levels of physical activity throughout the study. Physical activity level was assessed during the study using a 3-days (2 weekdays and 1 weekend day) physical activity questionnaire (data not shown).

### Composition of diets

The 2 experimental regimens were formulated so that the percentage of daily calories from fat (~29%), saturated fat (~10%), carbohydrates (~55%) and proteins (~17%) (Table 1) complied with the dietary recommendations from the NCEP for primary prevention of CVD [25]. The seven-day cyclic menus in the MILK diet and the NCEP control diet were identical in terms of foods, calories and macronutrient composition, with the exception that milk and any other forms of dairy products were formally excluded from the NCEP control diet. An example of a one-day menu is presented in the Additional file 1: Table S1. Differences in macronutrient composition attributable to milk in the MILK diet were compensated, in the milk-free NCEP diet, by adding animal protein (mostly red meat and white egg power), carbohydrates (mostly fruits) and lipids (mostly lard). We felt it was more appropriate to use animal rather than vegetable sources of saturated fat to match the two diets. Cholesterol intake when expressed per 2000 kcal intake was very similar between usual diet and the two experimental regimens (260–280 mg/2000 kcal, not shown). Overall, this allowed us to assess the impact of milk consumption per se and not in comparison with replacement control foods. On the other hand, between-diet differences in micronutrient intake attributable to milk (calcium, vitamin D) were not adjusted for. The glycemic index and glycemic load of the NCEP and MILK diets were calculated using existing databases [27, 28]. The breakfast meal represented approximately 20% of the daily energy intake whereas lunch and dinner each provided 40% of daily energy intake. As indicated above, 20% of daily calories came from 2% fat milk, corresponding to 3.2 servings of milk per 2000 kcal.

**Table 1:** **Composition of the experimental diets as well as baseline dietary habits of participants**

		Diets	
	Usual	NCEP	MILK
**Energy (kcal)**	2113.0 ± 677.9	2306.6 ± 210.5^a^	2319.9 ± 266.6^a^
**Protein (%kcal)**	17.6 ± 2.6	17.4	17.3
**Carbohydrates (%kcal)**	50.2 ± 5.8	55.5^a^	55.6^b^
**Fat (%kcal)**	33.2 ± 5.2	28.9^a^	29.6^b^
**Saturated (%kcal)**	12.0 ± 2.8	9.4^a^	9.8^b^
**Monounsaturated (%kcal)**	13.5 ± 2.5	12.0^a^	12.6
**Polyunsaturated (%kcal)**	5.2 ± 1.3	4.8	4.9
**Cholesterol (mg/d)**	266.1 ± 90.8	319.8 ± 29.2^a^	322.2 ± 37.0^a^
**Total fibers (g/d)**	21.3 ± 6.8	25.8 ± 2.4^a^	25.8 ± 3.0^a^
**Calcium (mg/d)**	1167.3 ± 500.8	445.9 ± 40.7^a^	1550.4 ± 178.2^a,b^
**Vitamin D (mcg/d)**	6.6 ± 3.0	3.7 ± 0.3^a^	12.2 ± 1.4^a,b^
**Alcohol (g/d)**	2.7 ± 3.1	0^a^	0^a^
**Glycemic Index**	ND	54.2	51.3
**Glycemic Load**	ND	146.7	135.7

Values are shown as mean ± SD. ND: not determined.

^a^Significantly different from the usual diet (P < 0.05).

^b^Significantly different from the NCEP diet (P < 0.05).

Differences between diets were tested by the MIXED procedure for repeated measurements.

### Risk factor assessment

Most cardiometabolic outcomes were measured at the beginning and at the end of each dietary phase except for glucose tolerance, VLDL apoB and apoA-I kinetics and surrogates of cholesterol homeostasis and of endothelial function, which were assessed only at the end of each intervention period. Waist circumference was measured midway between the lowest rib and the iliac crest using a standard tape measure [29]. Blood pressure (BP) was measured after a 5 min rest in the sitting position. It was measured on the right arm using a standard mercury sphygmomanometer and was computed as the mean of 3 readings each separated by a 3 min interval. The Korotkoff sound V was taken as the diastolic blood pressure. Mean arterial blood pressure (MAP) was calculated as the sum of diastolic + 1/3 (systolic minus diastolic) BP.

Fasting blood samples (12-h fast) were collected from an antecubital vein into evacuated tubes containing disodium EDTA at the beginning and at the end of each intervention period for the measurement of fasting blood lipids. Samples were then immediately centrifuged at 4°C for 10 min at 1500 × g to obtain plasma samples, which were then stored at 4°C until processed. Plasma lipid concentrations were measured as described earlier [30]. ApoB and apoA-I concentrations were measured by nephelometry [31]. Nondenaturing 2% to 16% polyacrylamide gradient gel electrophoresis was used to characterized LDL particle size using plasma stored at −80°C as described previously [32]. Inflammatory state was assessed by plasma C-reactive protein (CRP) and plasma adiponectin concentrations. CRP levels were measured by using a commercially available, highly-sensitive immunoassay with a monoclonal antibody coated with polystyrene particles (Behring Latex Enhanced on the Behring Nephelometer BN-100; Behring Diagnostic, Westwood, MA) [33] and plasma adiponectin by commercial ELISA kit (Linco Research Inc., St Charles, MO, USA). Endothelial function was assessed via the surrogate markers endothelin-1, vascular cell adhesion molecule 1 (VCAM-1), intercellular adhesion molecule 1(ICAM-1) and E-selectin [34]. These markers were measured using commercially available assays (R&D Systems, Inc., Minneapolis, MN, USA). Cholesterol homeostasis was assessed also by validated plasma surrogate markers of cholesterol absorption (β-sitosterol and campesterol) and cholesterol endogenous synthesis (lathosterol) as previously described [35]. Concentrations were expressed relative to plasma total cholesterol concentrations (× 10^2^ μmol/mmol of cholesterol of the same GC run) to correct for the differing number of lipoprotein acceptor particles [36].

A 75-g oral glucose tolerance test (OGTT) was performed by each participant in the morning after an overnight fast at the end of the two diets. Blood samples were collected in EDTA-containing tubes (Miles Pharmaceuticals, Rexdale, Ontario, Canada) through a venous catheter placed in an antecubital vein at −15, 0, 15, 30, 45, 60, 90, 120, 150, and 180 minutes at the end of each diets for the measurement of plasma glucose and insulin concentrations. Plasma glucose was measured enzymatically [37], whereas plasma insulin was measured by radioimmunoassay with polyethylene glycol separation [38]. The glucose and insulin areas under the curve (AUC) were calculated after the 180 minutes OGTT using the trapezoid method. The HOMA [39], Cederholm [40] and Matsuda [41] insulin sensitivity indices were calculated from the OGTT glucose and insulin data.

### VLDLapoB and ApoA-I kinetics

VLDLapoB and apoA-I kinetics were investigated at the end of the MILK diet and the NCEP diet in nine (n = 9) women in the constantly fed state using a primed constant infusion of [5,5,5-^2^H_3_]-l-leucine, as described previously [42]. VLDLapoB (d < 1.006 g/ml) and apoA-I (d < 1.25 g/mL) at various time points during the infusion were individually isolated from whole plasma by ultracentrifugation as described previously [43, 44]. Fractional catabolic rate (FCR) of VLDLapoB and apoA-I were individually determined by fitting the isotopic ratios over time to monoexponential functions, using the SAAM II software (University of Washington, Department of Bioengineering, Seattle, WA), as described previously [42]. VLDLapoB-100 enrichment plateau was used as the forcing function reflecting precursor pool enrichment. Absolute production rate (PR) was calculated (in mg/kg/d) using the formula:


Pool size was calculated as the plasma apo concentration (mg/L) multiplied by plasma volume (value fixed at 0.045 L/kg body weight).

### Sample size estimate

Previous dietary intervention studies have observed SD of the treatment effects on lipid risk factors to range from 100% to 200% of the treatment effect per se*,* the largest SD usually being related to TG levels [45]. We have shown that the SD of the change in plasma TG levels following a high MUFA diet consumed for 6–7 weeks under controlled conditions was approximately 150% larger than the diet-effect per se (−0.17% ± 25% mmol/l, P = 0.0064, N = 32 men) [46]. We have performed our power calculations using a fairly conservative SD of the treatment effect that was equivalent to 180% of the treatment effect per se. Under the above assumptions and with a final sample size of 30, the study could detect with a power of 83% at α = 0.05 clinically meaningful treatment effects in any of the primary and secondary outcomes. For example, estimates indicated that a clinically meaningful increase in TG levels of 20% ± 36% associated with milk consumption would be significant at P = 0.05 with a power = 83%. Any greater treatment effect or any smaller SD of treatment effect, the latter being likely to occur for many of the risk variables of interest, would be measured with greater statistical power. Based on the expected attrition rate (10%), we had planned to recruit 34 women to have a sample size of 30 available for final analyses. However, as mentioned above, we did not achieve this target. Twenty-nine women were randomized, 27 completed the protocol and 2 dropped-out.

### Statistical analysis

Data were analyzed using mixed models for repeated measures in SAS (version 9.2; SAS Institute Inc, Cary, NC, USA). The primary analysis used changes (post diet values vs. diet-specific baseline values) as the dependent variable. Diet (MILK vs. NCEP) and sequence were treated as fixed effects and subject was treated as random effect. Diet-specific baseline values were included in the mixed models as co-variables when possible (such data were not available for surrogate markers of cholesterol homeostasis and of endothelial function, apo A-I and apo B kinetics and OGTT variables, which were measured only post-intervention). As shown in the results’ section, body weight and waist circumference decreased significantly during both phase, with a slightly greater reduction after the NCEP diet. Thus, change in waist circumference with diet was included in all models as a covariate to account for this potential confounding effect. Using changes in BMI rather than changes in waist circumferences as a covariate had no impact on the results (not shown). Treatment by sequence effects were tested for each outcome as a covariate but were included in the final model only when significant. This was the case for body weight, BMI, total plasma cholesterol, apo A-I, total apo B, diastolic BP, mean BP and mean glucose levels. Values are reported as means ± SD, unless stated otherwise. VLDL-C and fasting insulin values were log_10_-transformed prior to statistical analysis to normalize their distribution. Three subjects were found to have CRP concentrations >10 mg/L at least once during the course of the study. According to Pearson *et al.* [47], CRP levels > 10 mg/L reflect infection or acute inflammation. Those individual data at this particular time point were treated as “missing” in the mixed models for this specific risk factor. Differences were considered significant at P < 0.05.

## Results

All women were overweight or obese when screened and had a waist circumference greater than the threshold for the MetS based on the NCEP-ATP III criteria [25] (≥88 cm) (Table 2). Based on the same criteria, low HDL-C, high TG, high BP and high plasma glucose concentrations were present in 63%, 46%, 89% and 13% of the participants respectively at screening. A total of 63% women had MetS.

**Table 2:** **Characteristics of the 27 postmenopausal women at the screening visit**

Variable		Range
**Age (yrs)**	57±- 5	(48-66)
**Body weight (kg)**	83.3 ± 10.6	(68.1-110.3)
**Waist circumference (cm)**	99.1 ± 7.3	(89.0-116.0)
**Body mass index (kg/m** ^**2**^ **)**	31.9 ± 3.5	(26.2-39.0)
**Total cholesterol (mmol/L)**	5.9 ± 1.0	(4.1-7.9)
**LDL-cholesterol (mmol/L)**	3.73 ± 0.84	(2.2-5.4)
**HDL-cholesterol (mmol/L)**	1.33 ± 0.25	(1.0-2.1)
**Triacylglycerol (mmol/L)**	1.82 ± 0.74	(0.9-2.6)
**Systolic blood pressure (mmHg)**	118.8 ± 13.5	(96.0-150.0)
**Diastolic blood pressure (mmHg)**	73.5 ± 6.9	(61.0-90.7)
**Fasting glucose (mmol/L)**	5.59 ± 0.46	(4.8-6.7)
**% with metabolic syndrome**	63%	

Values are shown as mean ± SD.

**Figure 1:**
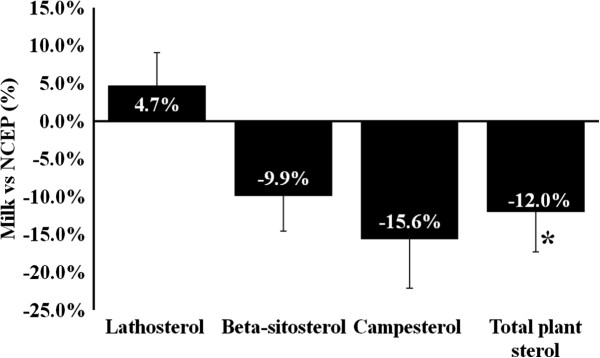
**Percent difference between diets in surrogate markers of cholesterol homeostasis.** *: Significant change, P < 0.05. Values are mean ± SEM. Differences between dietary phases were tested by the MIXED procedure for repeated measurements using post diet values as the dependent variable, with adjustment for post diet waist circumference. Similar results were obtained when values were reported as ratios to plasma cholesterol (not shown).

As shown in Table 3, consumption of the MILK diet for 6 weeks significantly reduced plasma HDL-C levels (−16.2%; P < 0.001) but had no impact on plasma total and LDL-C levels so that the total/HDL-C ratio was increased significantly compared with baseline values (+15.9%; P < 0.001). Changes in LDL-C and HDL-C after the MILK diet were similar in magnitude to the ones seen after the control NCEP diet (between-diet P > 0.05). On the other hand, the reduction in total plasma cholesterol concentrations was statistically more important after the NCEP diet than after the MILK diet (between-diet P = 0.01). Therefore, the total/HDL-C ratio increased in a similar extend during both phases (between-diet P = 0.26). Plasma levels of sterols (sum of beta-sitosterol and campesterol) were significantly lower at the end of the MILK diet than after the NCEP diet (−12.0%, P = 0.04, Figure 1) while no difference was seen between diets in plasma lathosterol concentrations.

**Table 3:** **Comparisons of the effects of the MILK and NCEP diets on the different cardiometabolic risk factors**

Variables	MILK				NCEP				P between
	Pre	Post	%	P within	Pre	Post	%	P within	
**Body weight (kg)**	82.6 ± 10.3	81.9 ± 10.4	−0.8%	0.003	82.8 ± 10.5	81.5 ± 10.4	−1.6%	<0.001	0.007
**Waist circumference (cm)**	102.7 ± 8.4	102.8 ± 7.9	0.1%	0.92	104.0 ± 9.0	101.8 ± 8.3	−2.1%	0.004	0.03
**Body mass index (kg/m** ^**2**^ **)**	31.7 ± 3.3	31.4 ± 3.4	−0.8%	0.001	31.7 ± 3.5	31.2 ± 3.4	−1.6%	<0.001	0.007
**Total cholesterol (mmol/L)**	5.75 ± 0.84	5.60 ± 0.87	−2.6%	0.39	5.87 ± 0.78	5.53 ± 0.76	−5.8%	<0.001	0.01
**VLDL cholesterol (mmol/L)** ^**a**^	0.55 ± 0.48	0.58 ± 0.43	5.3%	0.78	0.51 ± 0.32	0.47 ± 0.37	−7.9%	0.64	0.57
**LDL cholesterol (mmol/L)**	3.94 ± 0.55	3.97 ± 0.78	0.8%	0.96	4.11 ± 0.71	3.98 ± 0.71	−3.0%	0.36	0.42
**HDL cholesterol (mmol/L)**	1.27 ± 0.25	1.06 ± 0.19	−16.2%	<0.001	1.26 ± 0.20	1.08 ± 0.19	−14.1%	<0.001	0.29
**Total/HDL cholesterol ratio**	4.68 ± 1.10	5.42 ± 1.23	15.9%	<0.001	4.81 ± 1.12	5.28 ± 1.24	9.9%	<0.001	0.26
**Triglycerides (mmol/L)** ^**a**^	1.84 ± 0.86	1.87 ± 0.92	1.9%	0.77	1.78 ± 0.75	1.60 ± 0.78	−10.2%	0.14	0.14
**VLDL triglycerides (mmol/L)** ^**a**^	1.35 ± 0.78	1.40 ± 0.85	3.5%	0.64	1.29 ± 0.69	1.16 ± 0.73	−9.8%	0.25	0.18
**LDL size (Å)**	256.4 ± 2.8	256.3 ± 2.4	0.0%	0.64	256.1 ± 2.9	256.2 ± 2.6	0.0%	0.74	0.59
**LDL-PPD (Å)**	255.6 ± 2.9	255.3 ± 2.7	−0.1%	0.45	255.2 ± 3.0	255.5 ± 3.0	0.1%	0.59	0.27
**Apo A-I (g/L)**	1.52 ± 0.18	1.40 ± 0.16	−7.9%	<0.001	1.55 ± 0.17	1.36 ± 0.18	−12.1%	<0.001	0.06
**Apo B (g/L)**	1.11 ± 0.21	1.10 ± 0.22	−1.6%	0.29	1.13 ± 0.19	1.07 ± 0.18	−4.8%	0.02	0.37
**VLDL apo B (g/L)**	0.16 ± 0.08	0.16 ± 0.08	−0.5%	0.94	0.14 ± 0.06	0.13 ± 0.07	−7.2%	0.36	0.42
**LDL apo B (g/L)**	0.96 ± 0.15	0.94 ± 0.19	−1.8%	0.32	0.98 ± 0.17	0.94 ± 0.15	−4.4%	0.06	0.53
**CRP (mg/L)**	2.94 ± 1.80	3.19 ± 2.30	8.5%	0.40	3.38 ± 2.49	3.09 ± 2.33	−8.8%	0.21	0.15
**Adiponectine (mmol/L)**	10.52 ± 4.31	9.36 ± 4.26	−11.1%	0.01	10.80 ± 4.47	9.36 ± 4.09	−13.3%	0.001	0.58
**Endothelin (pg/mL)**	0.65 ± 0.25	0.57 ± 0.30	−11.5%	0.003	0.66 ± 0.35	0.55 ± 0.23	−17.6%	0.001	0.85
**VCAM-1 (ng/mL)**	nd	655.2 ± 179.4			nd	690.7 ± 184.5	−5.1%		0.25
**ICAM-1 (ng/mL)**	nd	203.8 ± 48.8			nd	193.9 ± 32.6	5.1%		0.22
**E-selectin (ng/mL)**	nd	32.8 ± 15.3			nd	30.5 ± 12.3	7.5%		0.51

Values are shown as mean ± SD.

^a^Values were log-transformed before statistical analyses.

Diet specific baseline values and changes in waist circumference were included as covariates in all models, except when outcomes were measured only post diet, in which case post diet waist circumference was included as a covariate. Change in waist circumference was also not included as a covariate when analysing this particular outcome. PPD: peak particle diameter, Apo: apolipoprotein, CRP: C-reactive protein, VCAM-1: vascular cell adhesion molecule 1, ICAM-1: Intercellular adhesion molecule 1, nd: not determined.

The reduction in plasma apo A-I concentrations after consumption of the MILK diet for 6 weeks (−7.9%, P < 0.001) compared to baseline values tended to be smaller than the reduction seen after NCEP (−12.1%, within-diet P < 0.001, between-diet P = 0.06). This difference could not be ascribed to differences in apo A-I PR or FCR between diets (Table 4). Neither the control NCEP nor the MILK diets had an impact on plasma total VLDL-C, VLDL-TG or VLDL-apoB concentrations, although there was a significant reduction in VLDL apoB FCR after the MILK diet compared with the NCEP diet (−13.4%; P = 0.04; Table 4). Neither the MILK nor the NCEP diets had an impact on measures of LDL particle size.

**Table 4:** **VLDL apoB and apoA-I kinetic data in a subsample of nine (n = 9) postmenopausal women after the MILK and NCEP diets**

Variables	MILK	NCEP	%	P
**Apo A-I**				
Pool size (mg)	5282 ± 892	4771 ± 885	10.7%	0.25
Production rate (mg/kg/d)	19.1 ± 3.1	17.6 ± 6.5	8.5	0.73
Fractional catabolic rate (pools/d)	0.29 ± 0.05	0.29 ± 0.09	1.3%	0.87
**VLDL apoB**				
Pool size (mg)	681 ± 287	605 ± 242	12.7%	0.08
Production rate (mg/kg/d)	36.0 ± 14.7	37.6 ± 13.5	−4.4%	0.45
Fractional catabolic rate (pools/d)	4.74 ± 1.81	5.47 ± 1.84	−13.4%	0.04

Values are mean ± SD.

Apo: apolipoprotein.

Differences between diets were tested using MIXED models for repeated measurements, while including waist circumference (post diet values) as a covariate.

**Figure 2:**
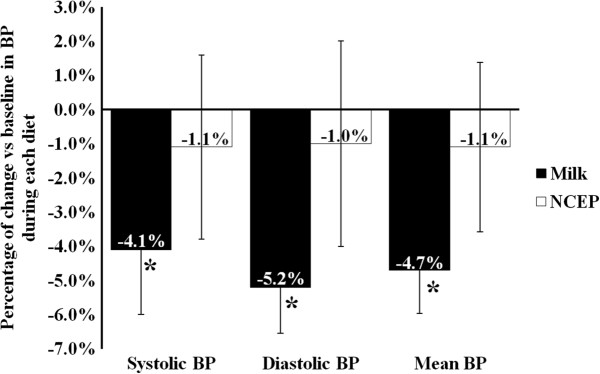
**Changes from baseline in systolic, diastolic and mean blood pressure (BP) after the MILK and the control NCEP diets.** Within and between-diet differences were tested by the MIXED procedure for repeated measurements using the change in blood pressures from baseline as dependent variables. *: Significant within-diet change (change from baseline, P < 0.05). Values are mean ± SEM. Diet-specific baseline values and changes in waist circumference were included as covariates in the models.

While there was no effect of the MILK and NCEP regimens on plasma CRP, both treatments reduced plasma adiponectin in a similar way (−11.1% and −13.3% respectively, between-diet P = 0.58). MILK significantly reduced systolic (−4.1%) and diastolic (−5.2%) BP compared with baseline values (Figure 2). Although smaller in magnitude and not significant after the NCEP diet, reductions in systolic and diastolic BP were comparable between the two treatments. The reduction in mean BP with MILK (−4.7%, P < 0.05) tended to be greater than after the NCEP diet (−1.1%, NCEP within-diet P > 0.05, between-diet P = 0.07). Plasma endothelin concentrations decrease significantly and similarly during both treatments (between-diet P = 0.85). Post diet levels of VCAM-1, ICAM-1 and E-selectin were similar between MILK and NCEP (Table 3).

Finally, both the MILK and NCEP diets similarly reduced plasma fasting glucose levels compared with diet-specific baseline values (between-diet P = 0.22, Table 5). There was no significant difference in any other markers of glucose homeostasis or any insulin sensitivity indices between treatments.

**Table 5:** **Comparisons of the effects of the MILK and NCEP diets of indices of insulin and glucose homeostasis**

Variables	MILK				NCEP				P between
	Pre	Post	%	P within	Pre	Post	%	P within	
**Fasting glucose (mmol/L)**	6.08 ± 0.39	5.77 ± 0.45	−5.1%	<0.001	5.98 ± 0.47	5.80 ± 0.53	-3.1%	0.009	0.22
**Fasting insulin (mmol/L)**	160.0 ± 93.5	159.9 ± 81.3	0.0%	0.96	166.6 ± 113.8	148.2 ± 91.2	−11.0%	0.11	0.09
**Glucose AUC (mmol/L/min)**	nd	1415 ± 57			nd	1437 ± 381	−1.5%		0.29
**Insulin AUC x10** ^**5**^ **(mmol/L/min)**	nd	1.96 ± 1.60			nd	2.03 ± 1.74	−3.3%		0.85
**ISI Cederholm**	nd	11.9 ± 4.0			nd	11.8 ± 4.6	0.3%		0.68
**ISI Matsuda**	nd	5.16 ± 2.66			nd	5.55 ± 3.49	−7.0%		0.31

Values are mean ± SD, N = 25 for all variables.

Differences between diets were tested using MIXED models for repeated measurements.

Diet specific baseline values and changes in waist circumference were included as covariates in all models, except when outcomes were measured only post diet, in which case post diet waist circumference was included as a covariate.

AUC: area under the curve, ISI: Insulin sensitivity index.

## Discussion

A systematic review published in 2011 concluded that the majority of available epidemiological studies suggest potential benefits of dairy consumption on the risk of having or developing MetS [48]. However, the authors have highlighted important methodological heterogeneity between studies and potential biases that prevent firm conclusions to be drawn. They have also emphasized on the importance of undertaking high quality randomized clinical trials to further assess the effect of individual dairy foods on cardiometabolic risk.

According to the recent meta-analysis of 20 randomized controlled studies by Benatar *et al.* [49], consumption of either high fat or low fat dairy has no impact on LDL-C, HDL-C, CRP, and on systolic and diastolic BP. The average difference in dairy intake between the dairy and control groups or treatments in these studies approximated 3 serving sizes /d. In these studies, the majority of participants (78%) were female, mean age was 51 ± 16 years and median duration of follow up was 26 weeks. Fifteen studies had a parallel group design and 5 were crossover studies. Only 1 of the 20 studies was a fully controlled feeding study and 4 were in postmenopausal women specifically. Authors have characterized most studies as being of moderate quality. The present study was carefully controlled and results are quite consistent with data from this meta-analysis. Indeed, we have shown that consumption of approximately 3 servings/day (3.2 servings/2000 kcal) of partly skimmed 2% fat milk in the context of an NCEP prudent diet has essentially no positive or negative impact on a wide spectrum of cardiometabolic features associated with MetS in postmenopausal women with abdominal obesity. On the other hand, our data and those from Benetar *et al.* [49] are mostly inconsistent with data from epidemiological studies, which have shown favorable relationships between milk intake and plasma HDL-C and TG concentrations [12, 50]. Such inconsistencies may be due to many factors, including important differences in the nutrient content of milk and dairy products investigated in these studies, but also more simply on inherent differences in study design and analyses between clinical trials and observational studies [51].

Data from randomized clinical studies meta-analysed in [49] indicated that intake of high fat dairy, not low fat dairy, increases plasma glucose concentrations. This apparent hyperglycemic effect of high fat dairy was largely attributed to one specific study, which accounted for 50% of the sample size in the analysis. The HOMA insulin sensitivity index was unchanged after dairy consumption (low and high fat not specified), although it is stressed that this assessment is based on only 4 studies [49]. In our study, consumption of 2% fat milk for 6 weeks in the context of a prudent diet reduced plasma glucose concentrations, but this was not more important than the reduction seen with the NCEP prudent diet alone. The impact of the 2 diets on plasma insulin levels and on the HOMA insulin sensitivity index was also very similar. In contrast with these data from clinical studies, consumption of total dairy and cheese, but not milk, has been associated in epidemiological studies with a reduced risk of type 2 diabetes [16–18]. This is in part consistent with results on glucose homeostasis from the present trial. However, the shorter term of clinical studies may not be sufficient to yield full beneficial effects of dairy products other than milk on glucose homeostasis and subsequently on the risk for type 2 diabetes. It is also possible that residual confounding associated with milk and dairy intake in epidemiological studies may result in favorable associations with type 2 diabetes. This apparent disconnect between clinical and epidemiological data needs further investigation.

Interestingly, plasma adiponectin levels decreased during both the MILK and the NCEP diets. Based on existing data [52], reductions in total fat and saturated fat intakes compared with usual intakes should have led to increased plasma adiponectin levels. Forbidding alcohol consumption during the MILK and NCEP diets may explain to some extent the reduction in adiponectin compared with baseline values. Indeed, moderate alcohol drinking had been associated with elevations in adiponectin levels [52]. The reduction in plasma HDL-C and apo A-I after both diets may also be attributable in part to the restricted alcohol consumption [53] as well as to reduced saturated fat intake [54] during the feeding phases compared with the participants’ usual diets. The kinetic sub-studies did not allow us to identify the exact mechanisms through which HDL-C and apo A-I were modified by milk consumption, nor why the reduction in plasma apo A-I tended to be smaller with the MILK diet compared with the control NCEP diet. Finally, there was no effect of milk consumption on plasma CRP concentrations in these postmenopausal women. Data from a recent systematic review by our group has shown that the impact of dairy on inflammation is not yet well characterized with inconsistent results between RCT [55].

Based on a recent meta-analysis of prospective population studies [15], each 200 g/d serving of milk is associated with a small but significant 4% reduction in the risk of hypertension. Consistent with these data, we have shown that 6-week consumption of 2% milk significantly reduced systolic, diastolic and mean blood pressure compared with baseline in postmenopausal women, who had in average relatively normal usual BP levels. However, only the reduction in mean blood pressure tended to be greater during the MILK diet compared with the control NCEP diet. Studies have suggested that certain peptides derived from milk proteins may modulate endothelin-1 release by endothelial cells, thereby partly explaining the anti-hypertensive effect of milk proteins [56]. Our data suggest that this pathway is probably not involved in the present case since the changes in plasma endothelin-1 concentrations were comparable between the 2 diets.

This study investigated the impact of milk consumption in the context of a prudent NCEP diet on a comprehensive list of cardiometabolic risk factors. Unlike previous studies that have combined various dairy products, results from the present study can be ascribed only to milk. Indeed, milk was replaced in the control diet by specific nutrients and not with control foods that might have confounding effects on their own. Although not conclusive, the study examined potential mechanisms underlying the changes in plasma lipids using surrogate markers of cholesterol metabolism and VLDL and HDL kinetics. The relatively small sample size may have limited our ability to detect changes in cardiometabolic risk factors. Studies have shown that periods as short as three days were sufficient to induce significant changes in cardiometabolic risk factors including plasma TG, LDL-C and small dense LDL [57]. We have also shown recently that blood pressure was modified after a 4-week period of a dairy rich diet in a partly controlled feeding context [58]. Nevertheless, we recognize that testing the impact of milk consumption over a longer period may amplify some of the minor changes seen in the present study.

## Conclusions

Data from this controlled-feeding study indicated that short-term consumption of 2% fat milk in the context of a low fat prudent diet has limited effects on a wide spectrum of cardiometabolic risk factors associated with MetS in postmenopausal women with abdominal obesity. Further studies are needed to explain why milk or dairy consumption in epidemiological studies is generally associated with reduced risk of MetS, CHD and type 2 diabetes.

## Electronic supplementary material

Additional file 1: Table S1: Typical one day menu on the MILK and the NCEP milk-free diets. (DOC 34 KB)

## Abbreviations

Apo: Apolipoprotein
AUC: Area under the curve
BP: Blood pressure
C: Cholesterol
CRP: C-reactive protein
CVD: Cardiovascular disease
FCR: Fractional catabolic rate
FFQ: Food frequency questionnaire
ISI: Insulin sensitivity index
ICAM-1: Intercellular adhesion molecule 1
MAP: Mean arterial blood pressure
MetS: Metabolic syndrome
NCEP ATPIII: National Cholesterol Education Program Adult Treatment Panel III
OGTT: Oral glucose tolerance test
PR: Production rate
TG: Triglycerides
VCAM-1: Vascular cell adhesion molecule 1.
