# Supplementary material for: Impact of milk consumption on cardiometabolic risk in postmenopausal women with abdominal obesity
Source: Nutr J. 2015 Jan 21;14:12. doi: 10.1186/1475-2891-14-12 (PMC4328687; doi:10.1186/1475-2891-14-12)
Supplement: Supplementary file 1 — Additional file 1: Table S1: Typical one day menu on the MILK and the NCEP milk-free diets. (DOC 34 KB) [file 12937_2014_871_MOESM1_ESM.doc]

**Additional files**

Supplemental TABLE 1. One day menu of the MILK and the NCEP milk-free diets

|  | **Tuesday** | |
| --- | --- | --- |
| **Meal** | **NCEP** | **Milk** |
| **Breakfast** | Bread  Fruit jam | Oat cereals |
|  | Margarine |  |
|  | Strawberry cocktail | Almonds |
|  | Peanuts | Milk |
| **Lunch** | Chili con carne | Chili con carne |
|  | Green salad and vinaigrette | Green salad and vinaigrette |
|  | Basmati rice | Basmati rice |
|  | Apple and grape cake | Apple and grape cake |
|  |  | Milk |
| **Dinner** | BBQ chicken | BBQ chicken |
|  | Peas and carots | Peas and carots |
|  | Potatoes  Bread margarine | Potatoes  Bread margarine |
|  | Brownies | Brownies |
| **Snack** | Bread + fruit jam | Milk |
